# Evaluation of the effect of periodontal health and orthodontic treatment on gingival recession: a cross-sectional study

**DOI:** 10.1186/s12903-025-06449-6

**Published:** 2025-07-02

**Authors:** İsmail Gül, Resul Çolak, Orhan Cicek

**Affiliations:** 1 Department of Periodontics, Faculty of Dentistry, Zonguldak Bülent Ecevit University, Esenköy/Kozlu, Zonguldak, 67600 Türkiye; 2 Department of Orthodontics, Faculty of Dentistry, Zonguldak Bülent Ecevit University, Esenköy/Kozlu, Zonguldak, 67600 Türkiye

**Keywords:** Gingival recession, Gingival phenotype, Orthodontic treatment, Periodontal health

## Abstract

**Background and aim:**

Periodontal health is a critical factor in the development of gingival recession, which may be influenced by orthodontic treatment and various patient-related factors. The aim of this study was to evaluate the prevalence of gingival recession observed during the retention phase after orthodontic treatment and the contributing etiological factors.

**Materials and methods:**

A total of 96 patients (65 females, 31 males; mean age 20.39 ± 2.21 years) were included in the study during routine follow-up examinations in the retention phase, at least six months after the completion of non-extraction fixed orthodontic treatment and their sociodemographic data, oral hygiene habits, and clinical periodontal measurements were evaluated. The relationships between dentoalveolar cephalometric measurements obtained from lateral cephalograms and gingival recession and gingival phenotype were evaluated. The normality of the data was assessed using the Kolmogorov-Smirnov test, and Mann-Whitney U and Chi-square tests were applied for continuous and categorical variables, respectively. Logistic regression analyses were performed to evaluate risk factors. Statistical significance was considered as *p* < 0.05.

**Results:**

Gingival recession was found to be more prevalent in thin gingival phenotypes compared to thick phenotypes and was observed to be more pronounced when bleeding on probing was 30% and higher (*p* < 0.05). It was observed that gingival recession increased with age (*p* < 0.05). No statistically significant difference was found between gingival recession and other periodontal clinical measurements, sociodemographic data, and oral hygiene habits (*p* > 0.05). No statistically significant relationship was observed between lower incisor protrusion and gingival recession (*p* > 0.05).

**Conclusions:**

It was concluded that (i) gingival phenotype, bleeding percentage on probing, and age had a considerable effect on gingival recession, whereas orthodontic tooth movement had no significant effect, and (ii) after orthodontic treatment, despite the achievement of a well-aligned teeth and dental arch, the frequency of periodontal check-ups should be increased to reduce the risk of gingival recession.

**Clinical relavance:**

Orthodontic treatment, consider periodontal conditions related to gingival recession, like thin phenotype and bleeding on probing.

## Introduction

In dental practice it is important to be aware of the factors that can influence the final aesthetic and functional outcome of a treatment. The result should be evaluated using a multidisciplinary approach that considers periodontal, restorative, prosthetic, and orthodontic factors. In addition, the correct identification and assessment of soft tissues are essential in decision-making [1–3].

Although various methods have been used to assess the gingival phenotype [4], the periodontal probing method is particularly noteworthy due to its widespread use, ease, and non-invasiveness [5]. The reflection of the periodontal probe indicates a thin phenotype of the gingiva, while non-reflection indicates a thick phenotype [6, 7]. The periodontal phenotype is critical for maintaining the stability of marginal soft tissue against factors such as inflammation and mechanical trauma that can affect both periodontal and peri-implant tissues [5]. Studies have shown that the risk of gingival recession is higher in thin gingival phenotypes than in thick gingival phenotypes [8–10].

Gingival recession, defined as apical migration of the cementoenamel junction, may cause several adverse outcomes, including root surface caries, aesthetic problems, dentin sensitivity, and, in advanced cases, tooth loss [11, 12]. A variety of factors, including age, gender, genetics, orthodontic tooth movements, gingival phenotype, incorrect brushing techniques, malocclusion, the presence of dehiscence or fenestrations in the alveolar bone, gingival infection, abnormal frenulum attachment, and iatrogenic factors, play a role in gingival recession [13].

While previous studies reported no association between incisor proclination resulting from orthodontic treatment and gingival recession [14–17], other studies have demonstrated a significant correlation between orthodontic treatment and gingival recession on the labial surface, indicating an approximate 0.2 mm increase in recession for each degree of increased incisor inclination [18–20]. The literature contains conflicting statements regarding the relationship between orthodontic forces and gingival recession [15–20]. Additionally, there are a limited number of studies evaluating the prevalence of gingival recession following orthodontic treatment, as well as the potential effect of orthodontics, demographic factors, and periodontal health on gingival recession [21, 22].

In recent years, the effects of orthodontic treatment on periodontal health and gingival recession have been a key area of research focus. However, the majority of studies have focussed on the period during or immediately after orthodontic treatment [8, 22–27]. On the other hand, Morris et al. [24] reported that the prevalence of gingival recession after orthodontic treatment was 5.8%, but this rate increased to 41.7% during the retention phase. Similarly, Renkema et al. [21] determined the prevalence of gingival recession after orthodontic treatment to be 6.6%; they observed that this rate increased to 20.2% within 2 years after treatment and to 37.7% after 5 years.

Gingival recession is commonly observed during the retention phase after orthodontic treatment. However, data on the etiopathogenesis of gingival recession observed in this period are limited. Studies examining the role of periodontal parameters, such as plaque index, gingival bleeding index and probing pocket depth, in this process are limited [9, 10].

The success of orthodontic treatment is closely linked to rigorous oral hygiene practices, both at the personal and professional levels, throughout all phases of therapy. Numerous studies have highlighted the significance of maintaining optimal oral hygiene before, during, and after orthodontic interventions, underscoring its critical role in preventing periodontal complications and ensuring favorable treatment outcomes [28, 29]. Given the growing demand for orthodontic treatment and the significance of maintaining periodontal health, it is crucial to assess the nature and extent of complications associated with these interventions in patients with different gingival phenotypes. To the best of our knowledge, despite conflicting reports regarding the relationship between orthodontic treatment and periodontal health, there are few studies that have addressed orthodontic treatment-related changes in marginal periodontal tissues [15, 17, 20]. This study investigates the relationship between periodontal health and orthodontic tooth movements and gingival recession, aiming to provide valuable insights into periodontal and orthodontic prognosis.

Therefore, the aim of this study was to evaluate the effect of gingival phenotype and periodontal health on gingival recession in patients referred to the periodontology clinic for routine follow-up during the post-orthodontic retention phase. Additionally, the relationship between angular and linear measurements on lateral cephalometric radiographs and gingival recession was investigated. The first H1 hypothesis of the study is that gingival phenotype and periodontal health have an effect on gingival recession. The second H1 hypothesis of the study is that angular and linear orthodontic measurements have an effect on gingival recession.

## Materials and methods

### Study design and ethical approval

This study included patients who applied to Zonguldak Bülent Ecevit University, Department of Periodontology for routine control during the retention phase after orthodontic treatment.

The study was designed as a single-center, cross-sectional study. Prior to the study, ethical approval was obtained from Zonguldak Bülent Ecevit University Non-Interventional Clinical Research Ethics Committee with the number 2023/14 − 6 on July 19, 2023. Written informed consent was obtained from all patients enrolled in our study in accordance with the Declaration of Helsinki.

### Sample and criteria

The required sample size was determined using G^*^Power software (version 3.1.9.7; Franz Faul, Kiel University, Kiel, Germany). An a priori power analysis was conducted with a two-sided Fisher’s exact test, setting α = 0.05 and statistical power (1-β) = 0.95. The analysis was based on the prevalence of gingival recession observed in the current study (p1 = 0.375) and the data from Melsen and Allais [8, 10], which reported a prevalence of 75.33% (p2 = 0.7533) with a precision of 10%. According to the sample size calculation, a total of 96 participants were required, with a calculated actual power of 0.954. The invitation to participate in the study was communicated to a total of 240 patients by telephone or face-to-face interview, and 96 of them agreed to participate in the study on a voluntary basis. Participation was entirely voluntary, and written informed consent was obtained from participants. In this context, the study employed a non-probability sampling method based on voluntary participation. In this context, a total of 96 patients were included in the study, and the inclusion criteria were as follows:

1. At least sixth month have passed since completed of non-extraction fixed orthodontic treatment (crowding and/or diastemas of 3 mm or less),
2. Patients aged 18 years or older.
3. Being systemically healthy or having a systemic disease under control,
4. No bad habits that may affect periodontal health (alcohol using, nail biting, thumb sucking, etc.),
5. Not being pregnant or in the lactation period,

Patients who did not meet at least one of these criteria were excluded from the study.

### Orthodontic interventions, radiograph acquisition, and cephalometric measurements

The study was based on the records of patients who underwent non-extracted fixed orthodontic treatment by specialists between 2018 and 2023 in the Department of Orthodontics, Faculty of Dentistry, Zonguldak Bülent Ecevit University. All subjects underwent fixed orthodontic therapy utilizing 0.022 × 0.028-inch MBT™ stainless steel brackets (Mini Master Series, American Orthodontics, Sheboygan, WI, USA) in accordance with the MBT™ prescription. The archwire sequence comprised an initial alignment phase with 0.012-inch, 0.014-inch, and 0.016-inch heat-activated nickel-titanium (NiTi) round wires, followed by a 0.016-inch stainless steel round wire. This was succeeded by the application of a 0.019 × 0.025-inch heat-activated NiTi rectangular wire and a final phase with a 0.019 × 0.025-inch stainless steel rectangular wire (American Orthodontics, Sheboygan, WI, USA) [30].

In the first phase of the study, pretreatment (T0) lateral cephalometric radiographs, panoramic radiographs, and intraoral photographs were retrieved for each patient by scanning the archive. Panoramic radiographs obtained from an X-ray machine (Veraviewepocs 2D, J Morita Mfg. Corp., Kyoto, Japan) were analyzed to determine whether there was increased resorption in the alveolar bone starting 2 mm apical to the enamel-cementum junction and whether orthodontic extraction had been performed. The sagittal position of the lower incisors at T0 was assessed from lateral cephalometric radiographs taken with the same X-ray machine, and cephalometric measurements (IMPA angle, L1-NB angle, L1-NB distance) were performed and recorded by a specialized orthodontist (OC). Cephalometric measurements were performed using cephalometric software (Nemoceph^®^ NX 2005, Nemotec, Madrid, Spain). The definition of the cephalometric measurements [31] is given in Table 1.

**Table 1:** Definitions of orthodontic angular and linear measurements

Orthodontic Parameters	
L1-NB angle:	The angle between the long axis of the lower central incisor, formed by the incisal edge and apex points, and the NB line (passing through the Nasion and B points).
Incisor Mandibular Plane Angle (IMPA) angle:	The angle between the mandibular plane (passing through the Gonion and Menton points) and the long axis of the lower central incisor.
L1-NB distance:	The sagittal distance from the most vestibular point of the lower central incisor crown to the NB line.

In the second phase, lateral cephalometric and panoramic radiographs during the earliest sixth-month follow-up of the retention period after orthodontic treatment (T1) were retrieved and analyzed. The IMPA angle, L1-NB angle, and L1-NB distance were re-measured on the obtained lateral cephalometric radiographs, and the difference between these measurements and the values measured at T0 was calculated and recorded. Panoramic radiographs were re-examined for any loss of teeth or alveolar bone during the retention phase.

### Periodontal examination

Periodontal examination, demographic records and oral hygiene habits of the patients who came to the periodontology clinic for routine control in the T1 period were recorded.

The age, gender, and smoking habits were recorded for the individuals included in the study. In addition, the frequency of tooth brushing (regular and irregular) [32], the use of dental floss, the duration of orthodontic treatment, the time elapsed after treatment, and frequency of dental visits after orthodontic treatment were inquired and recorded.

**Fig. 1:**
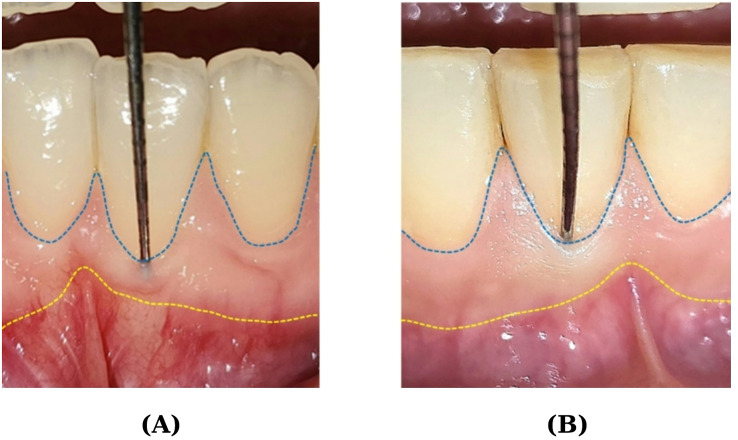
(**A**) Thin gingival phenotype visualized with a periodontal probe. (**B**) Thick gingival phenotype not visualized with a periodontal probe. The blue dashed line indicates the free gingival margin, and the yellow dashed line indicates the mucogingival junction

During the periodontal evaluation, the entire mouth was examined for:

- **Plaque Index (PI)**: Each tooth was scored in four areas: disto-buccal, mesio-buccal, mid-buccal, and mid-lingual/palatal, and the average was calculated [33, 34],
- **Gingival Index (GI)**: Each tooth was scored in four areas: disto-buccal, mesio-buccal, mid-buccal, and mid-lingual/palatal, and the average was calculated [35],
- **Presence of gingival recession**: Gingival recession was defined as ‘present’ if the distance of exposed root surface, measured from the enamel-cementum junction to the gingival margin, was more than 1 mm; if it was less than 1 mm, it was defined as ‘absent’. The presence of gingival recession was recorded at the patient level,
- **Bleeding on probing percentage**: Bleeding was assessed within 30 s of periodontal probing. The ratio of bleeding areas to all areas was calculated to obtain a percentage value. Based on the values obtained in studies measuring the degree of gingival inflammation, this value was recorded as (0) if it was below 30% and (1) if it was above 30% [36],
- **Probing depth (PD)**: The periodontal probe was placed into the gingival sulcus at four points of each tooth, and the distance between the pocket base and the gingival margin was measured and recorded as PD [37],
- **Keratinized gingival width (KGW)**: The length from the gingival at the mid-buccal point of the six anterior lower teeth to the muco-gingival junction was measured [38],
- **Gingival phenotype**: After placing the periodontal probe 1 mm into the gingival sulcus, it was checked whether the color of the probe was reflected by the gingiva. If the probe color was reflected, the gingiva was recorded as thin (Fig. 1A); if not, it was recorded as thick (Fig. 1B). The buccal surface of the lower right central incisor was used as a reference [39–41].
- **Frenulum stress**: The height of the frenulum attachment in the lower anterior region was evaluated for stress. If stress was present at the frenulum attachment point, it was recorded as ‘present’; if no stress was observed, it was recorded as ‘absent’ [42].

During these procedures, a UNC-15 periodontal probe (CPU 15 UNC, Hu-Friedy^®^, Chicago, IL, USA) was used. All periodontal parameters were recorded at the patient level, and all measurements were performed by the same examiner (IG), who was blinded to the participants’ orthodontic treatment history.

### Statistical analysis

SPSS package programme (Statistical Packet for Social Sciences, version 27.0 for Windows, Chicago, USA) was used for statistical analyses of the data obtained in the study. The normality distribution of the data was evaluated using the Kolmogorov-Smirnov test. Descriptive statistics of the data included minimum, maximum, median, mean and standard deviation values. Mann-Whitney U test was used for pairwise comparisons involving continuous variables and the Chi-square test for categorical variables. Univariate and multicategorical logistic regression analyses determined the relationship between dependent and independent variables. Binary logistic regression analysis was used to analyse the gingival recession risk factors. Gingival recession, accepted as the dependent variable for logistic regression analysis, was coded as 0 in its absence and 1 in its presence. In hierarchical regression models, patient-related factors and periodontal and orthodontic parameters were used as independent variables. Intra-class correlation coefficients (ICC, ‘Intra-class Correlation Coefficient) were used to evaluate the intraobserver reliability of repeated measurements at two-week intervals in 25% of randomly selected samples. The statistical significance level was set at *p* < 0.05.

## Results

For repeated measurements, high intra-observer reliability was found with ICCs ranging from 0.813 to 0.967 (*p* < 0.001).

A total of 96 volunteers (31 males, 65 females) were included in the study, with a mean age of 20.06 ± 2.08 years for males and 20.54 ± 2.27 years for females. Gingival recession was present in 36 patients (37.5%), and absent in 60 patients (62.5%). Descriptive data of the patients included in the study are presented in Table 2. Gingival recession greater than 1 mm in at least one tooth was observed in 36 patients (37.5%), whereas no gingival recession was detected in 60 patients (62.5%).

**Table 2:** Descriptive data of the patients included in the study

Variables			Sample (*n*)	Percentage (%)
Gender	Male	31		32.3
	Female	65		67.7
Cigarette	Not using	76		79.2
	Using	20		20.8
Presence of gingival recession	Present	36		37.5
	Absent	60		62.5
Frequency of tooth brushing	Irregular	17		17.7
	Regular	79		82.3
Dental floss use	Not using	76		79.2
	Using	20		20.8
Gingival phenotype	Thin	28		29.2
	Thick	68		70.8
Frenulum stress	Present	8		8.3
	Absent	88		91.7
Bleeding on probing (Full mouth)	≥%30	57		59.4
	<%30	39		40.6
Dentist visit	Regular	18		18.8
	Irregular	78		81.3
	**Median (Min.- Max.)**			**Mean ± Sd**
Age (years)	20 (18–27)			20.39 ± 2.21
Orthodontic treatment duration (month)	30 (6–84)			32.77 ± 17.29
Time after orthodontic treatment (months)	18 (6–60)			20.06 ± 12.19
Plaque index (Full mouth)	2.25 (0.25–2.75)			2.00 ± 0.72
Gingival index (Full mouth)	1.25 (0.25–2.50)			1.25 ± 0.81
KGW (mm)	3.08 (0.67–5.67)			3.09 ± 1.02
Probing depth	1 (1.40–2.60)			1.79 ± 0.30
L1-NB angle variance (°)	2 (-13–24)			2.25 ± 4.40
L1-NB distance variance (mm)	0.6 (-2.2–5.6)			0.74 ± 1.43
IMPA angle variance (°)	4 (-2–11)			4.63 ± 2.46

mm: millimeter Min.:minimum Max.:maximum SD: Standard Deviation KGW: Keratinized Gingival Width

**Fig. 2:**
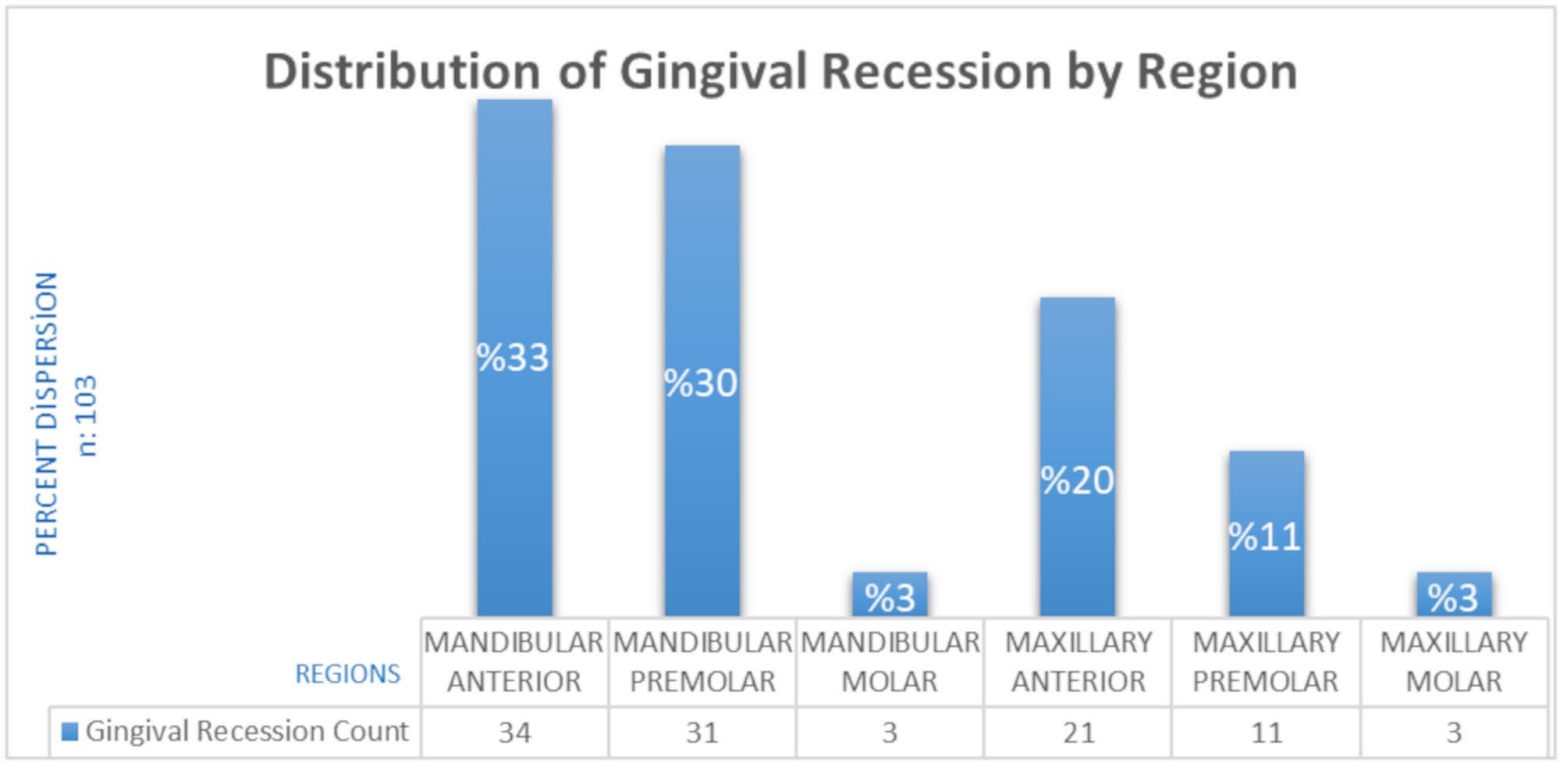
Distribution of gingival recession according to regions

A total of 103 teeth with gingival recession were identified in the study population of 96 patients. Gingival recession was observed more frequently in the mandible than in the maxilla, with the anterior region of the mandible being most affected. The regional distribution of the teeth with gingival recession is presented in Fig. 2.

Although gingival recession was more common in females than in males, the difference was not statistically significant (*p* > 0.05). Similarly, no statistically significant effect of smoking, oral hygiene habits, dental visits, duration of orthodontic treatment, and time elapsed following orthodontic treatment on gingival recession was observed (*p* > 0.05).

Gingival recession was observed in 68% of patients with a thin gingival phenotype compared to 25% of patients with a thick phenotype, and this difference was found to be statistically significant (*p* < 0.05).

A statistically significant relationship was found between increasing age of the patients included in the study and the presence of gingival recession (*p* < 0.05). In contrast, no statistically significant difference was found between the presence of gingival recession and SD, PI, GI, and KGW (*p* > 0.05). The statistical analysis results of the comparison of periodontal, orthodontic parameters, and patient-related factors based on the presence of gingival recession are presented in Table 3.

**Table 3:** Statistical analysis of the relationship between periodontal and orthodontic parameters and patient factors and the presence of gingival recession

Gingival Recession Presence		Present (*n*:36)	Absent (*n*:60)	*p*
Gender	Male (n:31)	10 (%32)	21 (%68)	0.464 ^x²^
	Female (n:65)	26 (%40)	39 (%60)	
Cigarette	Not using (n:76)	28 (%37)	48 (%63)	0.795 ^x²^
	Using (n:20)	8 (%40)	12 (%60)	
Frequency of tooth brushing	Irregular (n:17)	7 (%41)	10 (%59)	0.730 ^x²^
	Regular (n:79)	29 (%37)	50 (%63)	
Dental floss use	Using (n:20)	8 (%40)	12 (%60)	0.795 ^x²^
	Not using (n:76)	28 (%37)	48 (%63)	
Gingival phenotype	Thin (n:28)	19 (%68)	9 (%32)	**< 0.001** ^x²,*^
	Thick (n:68)	17 (%25)	51 (%75)	
Frenulum stress	Present (n:8)	5 (%62)	3 (%38)	0.147 ^f^
	Absent (n:88)	31 (%35)	57 (%65)	
Bleeding on probing (Full mouth)	≥%30 (n:57)	24 (%42)	33 (%58)	0.260 ^x²^
	<%30 (n:39)	12 (%31)	27 (%69)	
Dentist visit	Regular (n:18)	8 (%44)	10 (%56)	0.500 ^x²^
	Irregular (n:78)	28 (%36)	50 (%64)	
Orthodontic treatment duration (month)	6–18 month (n:27)	10 (%37)	17 (%63)	0.953 ^x²^
	≥ 19 month (n:69)	26 (%38)	43 (%62)	
Time after orthodontic treatment (months)	6–18 month (n:55)	19 (%35)	36 (%65)	0.489 ^x²^
	≥ 19 month (n:41)	17 (%41)	24 (%59)	
Age (years) Mean ± SD		21.42 ± 2.55	19.77 ± 1.73	**0.001** ^M, *^
Probing depth (mm) Mean ± SD		1.80 ± 0.28	1.79 ± 0.31	0.637 ^M^
Plaque index (Full mouth) Mean ± SD		2.00 ± 0.80	2.25 ± 0.70	0.623 ^M^
Gingival index (Full mouth) Mean ± SD		1.25 ± 0.75	1.50 ± 0.95	0.635 ^M^
KGW (mm) Mean ± SD		2.93 ± 1,03	3.18 ± 1,01	0.251 ^M^

x²: Pearson Chi-Square test, f: Fisher Exact Test, M: Mann Whitney U test, KGW: Keratinized Gingival Width, mm: millimeter, SD: Standard Deviation, ^*^: *p* < 0.05

The regression model (Model I) for the presence of gingival recession and patient-related factors was found to be statistically significant (*p* < 0.05).

A significant relationship was found between the presence of gingival recession and the age of the individuals, with gingival recession increasing 1.494-fold with increasing age (*p* < 0.05).

The regression model (Model II) for the presence of gingival recession and periodontal parameters was found to be statistically significant (*p* < 0.05).

A significant correlation was found between gingival phenotype and gingival recession (*p* < 0.05). Individuals with a thin gingival phenotype were 10.877-fold more prone to gingival recession than those with the thick phenotype, according to univariate logistic regression analysis (*p* < 0.05). Similarly, in multivariate logistic regression analysis, individuals with a thin gingival phenotype were 7.742-fold more likely to have gingival recession.

A statistically significant relationship was found between the percentage of bleeding on probing and gingival recession (*p* < 0.05). Patients with more than 30% bleeding when probing the entire mouth were found to have a 4.774-fold increased risk of gingival recession.

The regression model (Model III) of the presence of gingival recession and orthodontic parameters was not found to be statistically significant (*p* > 0.05). The statistical results of the binary logistic regression analysis of the factors affecting gingival recession are presented in Table 4.

**Table 4:** Investigation of the factors affecting gingival recession by binary logistic regression analysis

		Univariate Logistic Regression			Multiple Logistic Regression		
Gingival Recession Presence		OR	95% CI	*p*	OR	95% CI	*p*
**MODEL I (PATIENT-RELATED FACTORS)*p*** **= 0.021**							
Age		1.494	1.179–1.892	**< 0.001** ^*^	1.442	1.159–1.794	**0.001** ^*^
Gender	Male	References					
	Female	1.325	0.473–3.713	0.592			
Cigarette	Using	References					
	Not using	1.351	0.396–4.612	0.631			
Dentist visit	Irregular	References					
	Regular	1.03	0.308–3.450	0.961			
Frequency of tooth brushing	Regular	References					
	Irregular	2.213	0.644–7.604	0.207			
Dental floss use	Not using	References					
	Using	1.296	0.428–3.928	0.647			
**MODEL II (PERIODONTAL PARAMETERS)*p*** < 0.001^*^							
Gingival phenotype	Thick	References			References		
	Thin	10.877	3.077–38.455	**< 0.001** ^*^	7.742	2.753–21.770	**< 0.001** ^*^
Plaque index (Full mouth)		0.610	0.323–1.153	0.128			
Gingival index (Full mouth)		0.710	0.328–1.536	0.385			
KGW (mm)		1.181	0.696–2.002	0.537			
Probing depth		1.194	0.232–6.130	0.832			
Frenulum stress	Absent	References					
	Present	2.484	0.428–14.436	0.311			
Bleeding on probing (Full mouth)	<%30	References					
	≥%30	4.774	1.402–16.259	**0.012** ^*^			
**MODEL III (ORTHODONTIC PARAMETERS)***p* = 0.966							
Orthodontic treatment duration (month)		0.999	0.975–1.024	0.954			
Time after orthodontic treatment (months)		1.005	0,971-1,040	0.793			
L1-NB angle variance (°)		0.901	0.672–1.208	0.486			
L1-NB distance variance (mm)		1.133	0.474-2,707	0.778			
IMPA angle variance (°)		1.110	0.787–1.565	0.554			

OR: Odd Ratio (Coefficient), 95% Cl: 95% Confidence Interval, KGW: Keratinized Gingival Width, *: *p* < 0.05

Since some of the data collected in this study rely on participants’ self-reported information, it is possible that recall bias may have influenced the accuracy of the data. This potential bias could affect the strength and validity of the observed associations. Therefore, the results and their interpretation should be approached with caution, considering the possible impact of recall bias on the findings.

## Discussion

In light of the growing emphasis on oral and dental health, there is a pressing need for further research to understand the causes and consequences of gingival recession on overall health. Researchers have studied the causes, treatment methods, and preventive and protective treatments for gingival recession, which has a multifactorial etiology, for many years [34, 43, 44]. The starting point of our study was the evaluation of periodontal and orthodontic parameters together as factors affecting gingival recession in orthodontically treated patients.

It has been reported in a number of studies that the incidence of gingival recession increases with age [45–47]. The existing literature on the relationship between age and the presence of gingival recession in orthodontically treated patients is limited [8, 37, 48]. Ashfaq et al. [8], in their study investigating the effect of hard and soft tissue factors on gingival recession in orthodontically treated patients, found no statistically significant relationship between age and gingival recession. In contrast, Pernet et al. [48] found that the risk of gingival recession increased with age in their study of orthodontically treated patients. Similarly, in our study, we found that the presence of recession increased 1.494-fold with increasing age in orthodontically treated patients, and this increase was statistically significant (*p* < 0.05). This finding underscores the importance of increasing the frequency of periodontal follow-up visits after orthodontic treatment, with a minimum of once per year.

Gingival recession has been linked to poor oral hygiene in numerous studies, and a negative correlation has been identified between PI and gingival recession [49, 50]. The present study found no significant relationship between PI, GI, SD, and gingival recession. This finding may be due to the fact that our study population consisted of young individuals with high oral hygiene motivation.

Studies have shown that the percentage of bleeding on probing is an effective factor in gingival recession [40, 51]. In their histologic studies, Baker and Seymour [52] proposed that chronic gingival inflammation contributes to the development of gingival recession. In our study, a statistically significant correlation was found between the percentage of bleeding on probing and gingival recession, with a 4.774-fold increased risk of gingival recession in patients with bleeding above the %30 threshold (*p* < 0.05).

A variety of studies on gingival recession have shown that the prevalence of this condition varies depending on the type of tooth. Researchers have noted that gingival recession is most prevalent in the lower incisors [3, 46, 53]. Another study [54] noted that the upper first and second molars were the teeth most commonly affected by gingival recession. Böke et al. [39] reported in their study of orthodontically treated patients that canines were the most commonly affected teeth by gingival recession. Our findings suggest that, gingival recession was found to be more common in the lower jaw than in the upper jaw, with the lower central incisors in the anterior region being most commonly affected by gingival recession. The variation in outcomes across studies can be attributed to differences in age, oral hygiene habits, and dental treatment history of the study participants. In this cross-sectional investigation, the results were obtained and presented with a higher level of methodological standardization precision compared to other studies.

The growing interest in oral health is driving an increase in demand for orthodontic treatment, which can enhance both function and appearance [20]. However, the impact of incisor proclination or retroclination resulting from orthodontic treatment on gingival recession has been examined by numerous researchers, yielding conflicting results [8, 15, 24, 55]. Nastri et al. [15] found no statistically significant difference between incisor inclination and gingival recession. On the other hand, it has been reported that proclination of the incisors can lead to gingival recession, but it has also been noted that the clinical outcome of the minimal gingival recession found is doubtful [27, 56]. Ashfaq et al. [8], in their study of orthodontically treated patients without tooth extraction, reported a 33% prevalence of gingival recession in the anterior mandible; however, they concluded that incisor inclination was not a contributing factor to gingival recession. Our current results align with existing studies, indicating that linear and angular cephalometric measurements altered by orthodontic tooth movement do not have a statistically significant effect on gingival recession (*p* > 0.05).

Studies investigating the effect of orthodontic treatment duration on gingival recession have yielded different results. A systematic review on this topic [57] and the study by Khalil et al. [58] in orthodontically treated patients reported that there was no relationship between the duration of orthodontic treatment and gingival recession. Gebistorf et al. [59] found an increase in gingival recession with increasing age after orthodontic treatment. In a similar vein, Thomson [60] found gingival recession in 65.7% of patients eight years after completing orthodontic treatment. In our study, the mean treatment duration of the included patients was 2.73 years, while the mean post-treatment retention phase was recorded as 1.67 years. It was observed that neither the duration of orthodontic treatment nor the duration of the subsequent retention phase had a statistically significant effect on gingival recession.

The discrepancy between our findings and those reported in the literature may be attributable to the short-term effects resulting from the early retention phase of 1.67 years following orthodontic treatment. The findings of a relationship in other studies may be explained by the fact that these studies were based on long-term follow-up, as well as the cumulative effects of etiological factors such as age, oral hygiene habits, and smoking on gingival recession during this prolonged period. Given the potential for gingival recession to occur in the long term due to non-orthodontic factors, it is advisable to investigate whether gingival recession during the early retention phase of 1–2 years is associated with orthodontic treatment. In addition, waiting at least 6 months after orthodontic treatment for a clinical follow-up examination was considered important to allow the reversible infection of the gingival tissue to heal with plaque control and to avoid periodontal measurement errors [61].

There are many studies investigating the effect of gingival phenotype on gingival recession. Studies investigating gingival recession after orthodontic treatment have found a negative correlation between the gingival phenotype thickness and gingival recession [9, 15, 53, 62, 63]. In a similar study, Melsen and Allais examined 150 adult patients undergoing fixed orthodontic treatment without extraction and found a statistically significant correlation between the development or increase of gingival recession and gingival phenotype [10]. In contrast, another cross-sectional study evaluating gingival recession in 104 young adults found no statistically significant correlation between gingival phenotype and gingival recession [40]. Despite conflicting literature on the subject, our meticulously conducted study found a statistically significant relationship between gingival phenotype and gingival recession (*p* < 0.05). Our findings emphasize the importance of referring patients with a thin gingival phenotype to a periodontist for periodontal evaluation during or after orthodontic treatment.

One of the limitations of this study is that it included young adult patients and did not include other age groups. In this study, a non-probability sampling method based on voluntary participation was used. It should be kept in mind that this sampling method may have introduced selection bias. It is likely that the characteristics of individuals who declined to participate differ from those who accepted, which may limit the generalizability of the findings to the broader population. Therefore, this potential bias should be taken into consideration when interpreting the results. Another limitation is the inability to assess gingival phenotype and alveolar bone thickness prior to orthodontic treatment, as well as the lack of standardization of pretreatment malocclusion type and procedures like interproximal reduction, diastema, or crowding. Further studies with samples from a wider age range are needed to address these limitations. In the literature, the duration of orthodontic treatment has been reported between 17.56 ± 5.6 and 30.6 ± 8.0 months on average, although different results have been received in different studies [64–67]. It is thought that the prolongation in the duration of orthodontic treatment may be related to the fact that the start of treatment in some cases coincided with the COVID-19 pandemic and interruptions in follow-up processes.

The study revealed that individuals with a thin gingival phenotype exhibited a significantly higher risk of gingival recession during or after orthodontic treatment compared to those with a thick phenotype. Therefore, it is imperative to implement preventive measures prior to treatment, especially in individuals with a thin phenotype. Specifically, periodontal preparation supported by gingival grafts, preference for light force and minimal orthodontic tooth movement, and maintenance of optimal oral hygiene may be effective in preventing potential complications. These findings underscore the importance of a thorough periodontal evaluation and individualized preventive strategies prior to orthodontic treatment.

On the other hand, the method we used to determine the gingival phenotype is a current approach [64–66] and was performed by experienced researchers (İ.G. and R.Ç). Furthermore, the reliability of the results has been enhanced by ensuring researcher calibration. The Cohen’s Kappa value for the gingival phenotype was found to be 0.835, indicating a statistically significant (*p* < 0.001) and strongly positive correlation, demonstrating high reliability between measurements. In this respect, it is believed that our cross-sectional study makes a significant contribution to the limited literature where periodontal and orthodontic parameters are evaluated together, and it will also provide valuable clinical insights to clinicians regarding periodontal and orthodontic prognosis.

## Conclusions

Based on the findings and within the scope of this study, the following conclusions may be inferred:

- Gingival phenotype was found to be associated with gingival recession. In particular, individuals with a thin phenotype may be at increased risk of recession during both orthodontic treatment and the retention phase; therefore, this risk should be taken into consideration during treatment planning.
- A significant association was observed between increased age and bleeding on probing over 30% and gingival recession. These findings underscore the significance of a thorough periodontal evaluation and, when indicated, intervention during the post-orthodontic retention phase to prevent potential complications.
- However, no direct relationship was found between orthodontic treatment and gingival recession. This suggests that orthodontic treatment may not lead to gingival recession under certain periodontal conditions.

## Data Availability

The datasets used and/or analyzed during the current study are available from the corresponding author on reasonable request.

## Abbreviations

T0: Before orthodontic treatment (pretreatment)
T1: Retention period after orthodontic treatment
PI: Plaque index
GI: Gingival index
PD: Probing depth
KGW: Keratinized gingival width
OR: Odd ratio (coefficient)
